# Mitral Annular Plane Systolic Excursion (MAPSE) as a Predictor of Atrial Fibrillation Recurrence in Patients after Pulmonary Vein Isolation

**DOI:** 10.1155/2022/2746304

**Published:** 2022-09-27

**Authors:** Jan Alatic, David Suran, Damijan Vokac, Franjo Husam Naji

**Affiliations:** ^1^Department of Cardiology and Angiology, University Medical Centre Maribor, Maribor, Slovenia; ^2^Faculty of Medicine, University of Maribor, Maribor, Slovenia

## Abstract

**Introduction:**

Catheter ablation (CA) with pulmonary vein isolation (PVI) has become widely used in the past years for the treatment of atrial fibrillation (AF). Mitral annular plane systolic excursion (MAPSE) is the parameter that measures left ventricular longitudinal function, and it appears to be a good early marker of LV dysfunction. It is practically independent of poor image quality. The aim of our study was to analyse the role of echocardiographic variables, especially MAPSE in predicting the outcome of CA in patients with AF.

**Materials and Methods:**

We prospectively included 40 patients with paroxysmal and persistent AF that were referred for CA. All patients underwent radiofrequency CA with PVI. Standard transthoracic two-dimensional echocardiography was conducted one day after CA. Demographic data and the patients' characteristics were noted. The endpoint of our study was to estimate the AF recurrence rate diagnosed by ECG within 6 months of the follow-up period.

**Results:**

40 patients, mainly male (67.5%) with an average age of 61.43 ± 8.96 years were included in our study. The majority of patients had paroxysmal AF prior to ablation (77.5%). The AF recurrence rate was 20% after 6 months of follow-up. Lateral MAPSE in the AF-free group was greater than those who relapsed (1.57 ± 0.24 vs. 1.31 ± 0.25; *p* = 0.012). Patients who remained AF-free after a 6-month follow-up period had a significantly smaller left ventricular volume index (LAVI) than those who relapsed (34.29 ± 6.91 ml/m^2^ vs. 42.90 ± 8.43 ml/m^2^; *p* = 0.05). We found a significant reverse relationship between LAVI and MAPSE (*p* = 0.020).

**Conclusion:**

MAPSE and LAVI present risk factors for AF recurrence, specifically reduced MAPSE and larger LAVI, are related to AF recurrence after CA. In the future, MAPSE could play a significant role when predicting the CA outcome in patients with AF.

## 1. Introduction

Atrial fibrillation (AF) represents the most common cardiac arrhythmia worldwide [1–3]. It is associated with significant cardiovascular morbidity and mortality, and it produces immense health care costs. Future projections show a further increase in the prevalence of AF [4, 5]. A possible reason for the increasing prevalence might lie in the growing elderly population and/or a higher proportion of the population with other chronic diseases. Risk factors that predispose the development of AF are well described: age, coronary artery disease, hypertension, obesity, obstructive sleep apnoea, heart failure, and diabetes mellitus [6]. It is classified as paroxysmal, persistent, long-standing persistent, and permanent AF [7]. Current treatment recommendations consider rhythm control via pharmacological measures as highly significant. [8]. Furthermore, catheter ablation (CA) has become widely used in the past years with pulmonary vein isolation (PVI) being the cornerstone of the interventional treatment of AF. The success rate of AF ablation of paroxysmal AF is estimated at 70%, whereas in patients with persistent AF the estimation lies at 50% [9]. Currently, CA as a mode of treatment for AF is generally considered a second-line treatment [10]. Numerous predictors of CA have been previously discussed with data derived from echocardiography and cardiac magnetic resonance (CMR) studies [7, 11].

Echocardiography as a noninvasive diagnostic tool is vastly used for evaluating cardiac morphology and function [12]. Among the parameters for left ventricular (LV) function evaluation, mitral annular plane systolic excursion (MAPSE) is the parameter that correlates with LVEF [11]. MAPSE is evaluated by using M-mode and estimated by the extent of mitral annulus travel during the systole towards the apex and therefore assesses the global change in the size of the LV cavity [3, 13]. Moreover, it measures LV longitudinal function, which plays an important role in cardiac systolic function and it appears to be a good early marker of LV dysfunction, even before ejection fraction (EF) decline [13, 14]. Another MAPSE's valuable characteristic is that it is practically independent of poor image quality and sonographic windows as compared to biplane EF and speckle tracking evaluation [3, 11]. While the right ventricle dysfunction can affect the measurement of septal MAPSE, lateral MAPSE is more sensitive and specific for measuring the global longitudinal function of LV [3]. According to studies, MAPSE represents a strong predictor of the risk stratification and prognosis for patients with AF, heart failure, and postmyocardial infarction [3, 11, 13, 15]. Von Jeinsen showed that higher MAPSE is associated with a lower risk for all-cause mortality [15]. It also serves as a fair marker of structural abnormalities, such as myocardial fibrosis [3]. It has been also shown that lateral MAPSE measured by cardiac magnetic resonance (CMR) is an independent predictor of mortality in patients with hypertension [14].

The correlation between certain echocardiographic parameters and AF recurrence after CA is well implemented, specifically: atrial size, LVEF, LV diastolic dysfunction, LA strain, left atrial mechanical dyssynchrony, and functional mitral regurgitation [5, 16]. However, data on the role of MAPSE in AF and postablation prediction is scarce. When studying the correlation between MAPSE and AF, previous studies have only found a correlation with cardiac mortality implying that lower values of MAPSE are related to poor outcomes in AF patients [3]. To the best of our knowledge, the effect of MAPSE on the CA outcome in patients of AF is not known.

The aim of our study was to analyse the role of demographic and echocardiographic variables, especially MAPSE in predicting the outcome of CA in patients with AF.

## 2. Methods

### 2.1. Study Population and Protocol

We prospectively included 40 patients with paroxysmal and persistent AF that were referred to our institution for CA between 2018 and 2020. Patients aged 70 years and older, with established heart failure, low EF, and valve stenosis of any degree or more than mild valve regurgitation were excluded from our study. All patients underwent radiofrequency CA with PVI. Sex, age, body height, body weight, body mass index (BMI), and type of antiarrhythmic therapy (AAT) were noted.

Prior to ablation, a detailed bipolar voltage map of LA was obtained using a 20-polar catheter (Pentaray; Biosense Webster, Irvine, California). An automated 3D mapping system (Carto, Biosense Webster, Irvine, California) was used to construct the LA geometry and voltage map. To ensure the highest accuracy of the acquired atrial geometry by the 3D mapping system, respiratory gating was performed, and the atrial geometry was acquired at high adjustment settings. In addition, high-density mapping was added at sites where low voltage areas were recorded to define precisely the extent of the low voltage areas. Endocardial contact was ensured by fluoroscopy, electrogram stability, and the 3D mapping system. In our study patients underwent RFA in order to perform PVI with the aim to restore sinus rhythm. 24 hours before ablation the anticoagulation therapy was discontinued. Prior to ablation, transoesophageal echocardiography was performed in order to rule out the presence of a thrombus in the LA. The procedure was performed with patients awake and sedated. With the help of fluoroscopy transseptal puncture was performed. Ablation was done with 3.5-mm irrigated-tipped catheter (SmartTouch Thermocool, Biosense Webster, Irvine, California) followed by direct current cardioversion to restore sinus rhythm when needed. PVI was achieved with wide antral circumferential ablation. Mapping and CA were performed by one operator.

Standard transthoracic 2D echocardiography was performed one day after CA to obtain a standard recording of cardiac function and morphology. It was performed using the Vivid E95 station (General Electric Vingmed, Milwaukee, Wisconsin, USA). Two-dimensional (2D) and Doppler measurements were obtained according to recommendations for cardiac function and morphology quantification. Echocardiography was carried out with standard views and protocol and by one operator in order to eliminate interobserver variability. The following echocardiographic parameters were of special interest: biplane LA volume indexed to body surface area (BSA) (LAVI), lateral MAPSE by using M-mode, biplane EF (by Simpson method), diastolic function parameters (E, A by pulse wave Doppler, *e*', *a*', *s*' by Tissue Doppler (TDI)), tricuspid annular plane systolic excursion (TAPSE) by using M-mode.

### 2.2. Follow-Up

All patients had 12-lead ECG (25 mm/s, 10 mm/mV) recorded at the follow-up visit 6 months after CA to evaluate the basic rhythm. At the follow-up visit, patients were asked about the AF suggesting symptoms. Furthermore, they were also instructed to visit an outpatient clinic earlier in case of symptoms suggesting AF recurrence (dyspnoea, chest pain, dizziness, syncope, palpitations). The endpoint of our study was to estimate the AF recurrence rate diagnosed by ECG. Recurrence was therefore defined as documented AF within 6 months of the follow-up period.

### 2.3. Statistical Analysis

Data were analysed using SPSS version 26 (SPSS Inc., Chicago, IL, USA). Kolmogorov–Smirnov test was used to verify normal distribution. In the case of normally distributed continuous variables, data were presented as mean and standard deviation, whereas non-normally distributed continuous variables were expressed as median and interquartile range. Categorical data were summarised as frequencies and percentages. For comparisons of continuous variables, Student's *t*-test was used for normally distributed variables and the Mann–Whitney *U* test for non-normally distributed variables. For comparison of two categorical variables, Pearson-chi square test was used. In order to check the correlation between two continuous variables, Pearson's correlation test was used in the case of normally distributed variables, whereas for non-normally distributed variables and correlation between the categorical and continuous variables Spearman's correlation test was used. To predict how independent variables affect the dependent variable, binary logistic regression analysis was performed. For all tests, a two-tailed *p* value of ≤ 0.05 was considered statistically significant.

## 3. Results and Discussion

### 3.1. Results

In our prospective study, we included 40 patients, mainly male (67.5%) with an average age of 61.43 ± 8.96 years. The majority of patients had paroxysmal AF prior to ablation (77.5%). AAT was continued in the majority of patients after the CA (84.2%). The AF recurrence rate was 20% after 6 months of follow-up. All patients with AF recurrence were detected during the follow-up visit when an ECG was recorded. Furthermore, at the follow-up visit, two patients reported palpitations, and one of them went to the emergency room prior to the follow-up visit. Both presented with AF at the follow-up visit. Other demographic data are summarised in Table 1.

#### 3.1.1. The Impact of Patients' Characteristics on AF Recurrence Rate after CA

Those who were AF-free after a follow-up period were slightly heavier than those who relapsed (92.72 ± 15.08 kg vs. 92.50 ± 13.06 kg), but the difference was not significant (*p*=0.970). However, BMI was lower in the group of patients who remained AF-free (30.5 ± 4.31 kg/cm^2^ vs. 31.36 ± 5.59 kg/cm^2^), but the difference did not meet statistical significance either (*p*=0.636). Among those who had paroxysmal AF (77.5%), 83.9% remained AF-free after a follow-up period compared to the nonparoxysmal AF group of patients, of whom 71.4% remained AF-free. However, the difference was not statistically significant (*χ*^2^(1) = 0.588; *p*=0.443). 83.3% of those who remained AF-free after a 6-month follow-up continued with AAT, whereas 81.3% of those who had AF noted after a follow-up period were also on AAT after the procedure, the difference was not statistically significant (*χ*^2^(1) = 0.015; *p*=0.904).

#### 3.1.2. Relationship between Echocardiographic Parameters and AF Recurrence after CA

We found a statistically significant difference in lateral MAPSE values when comparing groups of patients who remained AF-free after a follow-up period than those who did not. Lateral MAPSE in the AF-free group was greater than those who relapsed (1.57 ± 0.24 vs. 1.31 ± 0.25; *p*=0.012). The difference in EF was not significant between the groups of patients who remained AF-free compared to those who relapsed (60% (IQR 0) vs. 60% (IQR 10); *p*=0.112). Patients who remained AF-free after a 6-month follow-up period had significantly smaller LAVI than the group where AF was noted at follow-up (34.29 ± 6.91 ml/m^2^ vs. 42.90 ± 8.43 ml/m^2^; *p*=0.05). A diastolic parameter *E*/*e*' was lower in the group of patients who remained in sinus rhythm than those who did not, but the difference did not meet a significant difference (8.25 (IQR 3.0) vs. 11.30 (IQR 8.3); *p*=0.193). The results are summarised in Table 2.

#### 3.1.3. Relationship between Other Variables and MAPSE

We investigated whether there is a correlation between demographic variables and MAPSE and we did not find any significant correlation between the variables. When investigating the relationship between MAPSE and other echocardiographic variables we only found a significant correlation between LAVI and MAPSE, being in reverse relationship to each other (*p*=0.020). Additionally, we performed logistic regression analysis in order to adjust MAPSE onto LAVI, and in this case, MAPSE failed to reach a point of significance (*p*=0.164).

### 3.2. Discussion

In our study, we demonstrated that MAPSE is significantly related to CA outcomes in patients with AF specifically reduced MAPSE correlates with AF recurrence after CA. To the best of our knowledge, there is no study that would test this hypothesis and its relation. According to the available literature, the authors showed that reduced MAPSE values are present in hypertensive patients. That might be due to the microcirculatory disturbances in the subendocardium of the LV that in turn leads to the reduction of longitudinal myocardial contraction force which translates into reduced MAPSE. Additionally, higher afterload due to hypertension imposes greater stress on the wall, which leads to a larger radius of curvature of the longitudinally directed fibres [3, 17, 18]. This was true regardless of the EF [3]. One possible explanation might be that early longitudinal impairment is compensated by augmented radial function [3, 13]. Romano et al. showed that MAPSE derived by CMR presents an independent predictor of mortality [14]. Subendocardium is more prone to injury due to the pathological disturbances stated above, which in turn leads to greater stress and higher oxygen consumption. Since myocardial fibres in the subendocardium are aligned more in the longitudinal direction, the earliest myocardial dysfunction leads to a reduction of mitral annular motion, which is exactly what is measured by MAPSE [14]. Secondly, reduced MAPSE was found in patients with aortic stenosis (AS), especially in symptomatic AS [3]. Authors showed that MAPSE is an independent predictor for the degree of AS, and is a more sensitive marker than LVEF [3, 19]. The mechanism responsible for the reduction in MAPSE is, similar to hypertension, higher wall stress, and intraventricular pressure that in turn lead to LV mechanical remodelling and eventually to subendocardial ischaemia and fibrosis [3]. Thirdly, reduced MAPSE was found in acute myocardial infarction patients and in patients with diastolic heart failure. Impaired LV relaxation and filling, and even systolic dysfunction due to normal ageing and increase in LV mass may lead to impairment of longitudinal fibres function that leads to restriction of mitral annular motion and therefore to reduced MAPSE [3, 13]. Finally, brain natriuretic peptide (BNP) levels correlate with worsening MAPSE [13, 20].

Interestingly, we found that reduced MAPSE is related to worse CA outcomes even though both tested groups had comparable EF. Furthermore, EF was preserved in both groups. EF is in fact a parameter of the radial function. The reason behind this probably lies in the aforementioned mechanisms that longitudinal function is impaired first, whereas other fibres compensate under pathological circumstances or due to ageing and therefore keep EF preserved until later stages [3].

According to the literature, two-thirds of people with AF develop HF, whereby HF with preserved EF (HFpEF) is more commonly observed in these cohorts of patients [21]. Mechanisms for cardiac mechanical and electrical remodelling are complex and still under investigation. However, remodelling manifests itself as changes in the cardiac size, shape and function in response to ageing, cardiac impairment, and increased load [21, 22]. The main mechanisms by which AF leads to pathological changes in the cardiac muscle are as follows: (i) loss of atrial systole and irregularity, (ii) tachycardia, (iii) decreased ventricular filling time, and (iv) diffuse fibrosis [8, 21]. They lead to mechanoelectrical and neurohormonal changes that eventually to adverse remodellation and impaired LV function that is expressed as increased end-diastolic pressure and volume with diastolic and systolic dysfunction [21]. Especially in short-term diastolic function is compromised first, and this is responsible for the injury of longitudinal fibres as mentioned above, and it can be expressed in reduced MAPSE even before EF declines [3, 8]. Here proposed mechanisms by which AF could affect MAPSE could therefore mean that MAPSE may be a result of irreversible changes in the myocardium done by AF per se. That would explain why reduced MAPSE is related to the worse outcome of CA in these patients. In the future, it would be interesting to see whether MAPSE could present a diagnostic criterion when considering CA in patients with AF.

Besides the significant difference in MAPSE when comparing CA outcomes, we also found that patients who had greater LAVI are less likely to remain in sinus rhythm after CA. This is in line with other available studies [16, 23–25]. Mano et al. showed that LA volume was larger in patients with AF recurrence mainly due to the reason that larger LA indicates advanced LA remodelling [23]. For example, Njoku et al. proposed in their meta-analysis that there is a 3% increase in the odds of AF recurrence per unit increase in LAVI [24]. LA remodelling is mainly expressed as structural, as well as electrical remodelling, which is the consequence of inflammation and interstitial fibrosis [24]. These eventually lead to new focuses of AF initiation that are within LA and are not limited to pulmonary veins only [24]. However, it is not known whether greater LAVI is a cause or a consequence of AF.

When reviewing the literature, markers that are related to AF recurrence after CA are LA size, impaired glomerular filtration rate (GFR), duration of AF, heart failure and LVEF, LV diastolic dysfunction, LA mechanical dyssynchrony, LA strain, functional mitral regurgitation, and age [5, 16, 25]. With our findings, we propose that MAPSE as a marker of longitudinal function of LV that is easily obtained regardless of the image quality could also play as a marker of AF recurrence after CA and could therefore become a part of the routine diagnostic workup of patients with AF and present a criterion in decision making for CA in patients with AF. Larger studies are needed to evaluate the significance of MAPSE in patients with AF undergoing CA and to determine the cut-off value at which there would be greater chances of AF recurring. Finally, it would be interesting to study the mechanism by which AF affects the longitudinal function and therefore MAPSE.

#### 3.2.1. Study Limitations

A relatively small number of patients and a short follow-up period present the main limitations of our study. Since there can be variability of MAPSE due to cardiac size, we should normalise our MAPSE measurements to heart size when performing echocardiography. It would also be useful to compare MAPSE measurement derived by TTE with MAPSE derived by CMR to estimate the role of MAPSE as a prognostic marker in AF patients after CA. Furthermore, either Holter monitoring or implanted loop recorder would be a more accurate method to assess the baseline cardiac rhythm during the follow-up period. Telemedicine would also be an interesting method to follow such patients. Finally, we did not adjust our findings regarding MAPSE to patients' comorbidities when analysing the data. Larger studies with longer follow-up periods are necessary to evaluate our findings.

## 4. Conclusion

In patients with AF undergoing CA MAPSE and LAVI present risk factors for AF recurrence, specifically reduced MAPSE and larger LAVI are related to AF recurrence after CA. In the future, MAPSE could therefore play a significant role when predicting the CA outcome in patients with AF.

## Figures and Tables

**Table 1:** Patients' characteristics.

Parameter	Mean and standard deviation
Age (years)	61.43 ± 8.96
Body height (cm)	173.47 ± 9.12
Body weight (kg)	92.67 ± 14.54
BMI (kg/cm^2^)	30.67 ± 4.52

List of abbreviations: BMI (body mass index).

**Table 2:** Relationship between echocardiographic parameters and AF recurrence after CA.

Parameter	AF-free survival after a 6-month follow-up	AF recurrence after a 6-month follow-up
MAPSE	1.57 ± 0.24	1.31 ± 0.25
EF (%)	60 (IQR 0)	60 (IQR 10)
LAVI (ml/m^2^)	34.29 ± 6.91	42.90 ± 8.43
*E*/*e*'	8.25 (IQR 3.0)	11.30 (IQR 8.3)

List of abbreviations: MAPSE (mitral annular plane systolic excursion); EF (ejection fraction); LAVI (left atrial volume indexed to body surface area).

## Data Availability

The data are not publicly available due to privacy and ethical concerns.
